# Gut microbiota and stress ulcers: unraveling the neurotransmitter connection

**DOI:** 10.3389/fnins.2025.1594179

**Published:** 2025-07-10

**Authors:** Khaled A. Abdel-Sater, Heba A. Hassan

**Affiliations:** ^1^Department of Dental and Medical Sciences, Faculty of Dentistry, Mutah University, Al-Karak, Jordan; ^2^Department of Clinical Pharmacology, Faculty of Medicine, Mutah University, Al-Karak, Jordan

**Keywords:** stress ulcer, microbiota-brain-gut axis, neurotransmitters, SCFAs, glutamate

## Abstract

**Background:**

Neurotransmitters are key signaling molecules in the brain-gut axis, may be involved in stress-induced ulcer damage.

**Objective:**

This study aims to address the relationship between gut microbiota and the stress response is examined in this review, with a focus on how neurotransmitters moderate the impact of stress on mucosal integrity and gut health. Understanding these mechanisms may open new avenues for therapeutic interventions targeting the brain-gut axis.

**Methods:**

A literature search was completed using PubMed, Web of Science, Cochrane, PsycINFO, Scopus and Embase databases for clinical and preclinical studies related to stress ulcer, gut microbiota and gut brain axis that published in English until November 2024.

**Results:**

The gut microbiota plays important role in preserving the health of the digestive system and influencing the body’s stress response through various pathways, including the enteric and autonomic nervous systems. This results in the production of microbial metabolites such as bile acids, tryptophan, and short-chain fatty acids that enter the bloodstream and go to the brain. Microbial neurotransmitters change the brain’s gastrointestinal axis.

**Conclusion:**

The connection between stress ulcer, neurotransmitters, and the gut microbiota was outlined in this review.

## Introduction

Gut microbiota is important for many the brain and gastrointestinal tract functions such as regulation of the food digestion, energy metabolism, inflammatory response, systemic immunity, intestinal motility, nutrient absorption, memory and learning (Cristofori et al., 2021). The brain-gut axis, is a communication network between the brain and gastrointestinal tract in both directions. The autonomic and enteric nervous systems, endocrine system, immune system, and neurotransmitters cooperated to regulate digestion, cognition, learning and anxiety (Góralczyk-Bińkowska et al., 2022). Dysbiosis refers to the disruption of the microbial community, which results in an imbalance of pathogenic species over helpful ones and disruption of the brain-gut axis and, alter stress response pathways (Góralczyk-Bińkowska et al., 2022). This dysfunction can exacerbate psychological stress and lead to increased gastric acid secretion, further contributing to ulcer formation (Karl et al., 2018). Normally, there is a balance between protective mucosal defense factors (such as the mucus barrier, bicarbonate secretion, prostaglandins, normal blood flow, cell renewal, endogenous antioxidants, and nitric oxide) and damaging factors (such as *Helicobacter pylori* infection, acid plus pepsin, stress, free radicals, nonsteroidal anti-inflammatory drugs, smoking, and alcoholism). Peptic ulcers develop when damaging factors such as *Helicobacter pylori* infection, acid, pepsin, etc. overcome protective mechanisms (Işık et al., 2024). It is the most common gastrointestinal tract disease and a serious medical problem that causes morbidity and mortality despite therapeutic intervention (Périco et al., 2020). Stress ulcers are acute mucosal lesions that occur in the gastrointestinal tract, primarily in the stomach and duodenum, owing to physiological stress (Karl et al., 2018). Stressors may include sepsis, shock, serious bacterial infections, burns (≥35% of the total body surface), trauma, organ failure and following surgery or psychological stress (Işık et al., 2024). The development of stress ulcers can be explained through several interrelated mechanisms; reduced gut blood flow may be due to hypotension or vasoconstriction, increased permeability of the gastrointestinal epithelium, decreased mucosal proliferation, decreased bicarbonate output in the stomach and duodenum, and increased reactive oxygen species (Popovic et al., 2023). Stress may increase the secretion of hydrochloric acid and pepsin, damaging the gastric mucosa (Martínez-Augustín et al., 2000). Stress may affect protective processes such as bicarbonate and mucus productions. Because hydrogen ions infiltrate an epithelium that has been made more permeable by ischemia, decreased bicarbonate secretion permits stomach acid to harm the epithelium, leading to intramural acidosis, ulceration, and cell death (Jia et al., 2023). Theoretically, bile reflux and mucosal barrier failure can be facilitated by decreased stomach motility (Işık et al., 2024). Stress-induced neurohormonal and sympathetic nervous system activation results in decreased stomach motility, gastric blood flow, and bicarbonate secretion (Martinou et al., 2022). The exact relationship between stress ulcers and the gut microbiota remains unclear. According to the hypothesis of this review gut microbiota homeostasis is crucial for preventing and treating stress ulcers especially through neurotransmitter activity. Therefore, this review was designed to understand the relationship between gut bacteria, stress response neurotransmitters, and stress ulcer.

## Role of microbiota-brain-gut axis in prevention stress ulcer

Ulcer gastric injury in critically ill patients can significantly influence the microbiota of the small intestine through mechanisms such as altered gastric acid secretion, ischemia–reperfusion injury, and increased intestinal permeability. Studies have shown that stress-induced changes in the stomach, including increase acid production and mucosal damage, promote dysbiosis in the small intestine, further compromising gut integrity and immune function (Martínez-Augustín et al., 2000).

### Maintains the gastrointestinal tract mucosal integrity

One of the key mechanisms by which gut microbiota influence stress ulcers is through the management of the function of the gut barrier. Gut microbiota is produced by modulating immunity, producing short-chain fatty acids (SCFAs), and influencing the production of mucous and antimicrobial peptides (Li et al., 2024). *Lactobacillus* and *Bifidobacterium* species, examples of beneficial bacteria, help in creating a protective barrier that shields the mucosal lining from pathogens and inflammatory stimuli (Tremblay et al., 2021). Increased permeability, or “leaky gut,” can result from disruption of the gut barrier. This increases the risk of mucosal damage and ulceration by enabling toxins and bacteria to enter circulation and intensify the inflammatory response. An imbalance in intestinal flora might hinder the regeneration and repair of mucosal tissue (Talarico et al., 2024).

### Immune activation

The gut—an important immunological organ—serves as a barrier to defend against infections coming from the outside and the biological environment inside (Talarico et al., 2024). Dysbiotic bacteria can induce the release of cytokines from immunological cells, which play a role in stress-induced mucosal damage (Houser and Tansey, 2017). Research has demonstrated that stress can change the gut’s pro-inflammatory and anti-inflammatory cytokine balance, resulting in elevated levels of cytokines, such interleukin (IL-6), TNF-α, and IL-1β (Tremblay et al., 2021). These cytokines may cause the gut barrier to malfunction, promote mucosal inflammation, and contribute to the development of gastrointestinal tract disorders (Wu et al., 2023). This inflammation can be exacerbated by dysbiosis, leading to an increased vulnerability to ulceration. Dysbiotic bacteria can also produce toxins that contribute to inflammation and mucosal injury, thereby promoting ulcer formation (Houser and Tansey, 2017).

### Microbial metabolites

Microbial metabolites such as SCFAs, tryptophan, and bile acid (BA) can pass through the blood–brain barrier (BBB). SCFAs are created when gut bacteria ferment dietary fibers and are exhibit anti-inflammatory and protective properties in the gut mucosa. Studies have shown that stress can alter the production of SCFAs in the gut, leading to dysbiosis and impairment of mucosal integrity (Tremblay et al., 2021). Dysbiosis can reduce the production of SCFAs, compromising mucosal repair and increasing vulnerability to ulceration (Li et al., 2024).

SCFAs are necessary for microglial proliferation and the integrity of the blood–brain barrier (Iftikhar et al., 2020) and promote the release of noradrenaline (NE), dopamine, serotonin (5-HT), and neuropeptide Y (NPY), all of which further control neuro-inflammation (Talarico et al., 2024). By controlling the synthesis of SCFAs and antioxidant enzymes, the gut microbiota plays a critical role in protecting against excessive oxidative stress (Sun et al., 2024).

A necessary amino acid, tryptophan is a building block of several physiologically active substances, including the neurotransmitter 5-HT (Wang et al., 2020). Tryptamine and indoles are two of the many tryptophan metabolites produced by gut microbiota (Rothhammer et al., 2018). This can affect astrocyte transcriptional programs and reduce central nervous system inflammation (Kennedy et al., 2017). Indoles control neuronal growth, differentiation, and neurodepressive-like effects on behavior (Kaur et al., 2019).

The liver and brain produce BA that can cross the blood–brain barrier and influences cognition, memory, and motor skills (Han et al., 2021). BA contributes to the regulation of cortisol production by inhibiting corticotropin-releasing hormone (CRH) release (McMillin and DeMorrow, 2016).

### Microbial neurotransmitters

There are two categories of neurotransmitters: small-molecule neurotransmitters and large-molecule neuromodulators. Small-molecule neurotransmitters include monoamines (epinephrine, NE, dopamine, and 5-HT), amino acids, glucocorticoids, and ACh. Large-molecule neuromodulators include neuropeptides, such as CRH, orexin, vasoactive intestinal peptide (VIP), and substance P. The need for multiple mediators in the stress response system arises from the complexity and variability of stressors encountered by organisms (Teleanu et al., 2022). Dysregulation of these neurotransmitters due to stress can impair gut function, increase gastric acid secretion, and contribute to ulcer development (Strandwitz, 2018).

#### Monoamines

Intestinal bacteria create dopamine through the enzymatic activity of aromatic amino acid decarboxylase (Liu et al., 2021). Most of the peripheral dopamine is produced from the gut, and gut bacteria can control peripheral dopamine levels (Khoder et al., 2016).

Epinephrine and NE are involved in rapid stress response. Epinephrine ensures an adequate energy supply through glycogen and fatty acid mobilization, while NE is vital for maintaining sympathetic tone and behavioral responses to stress (Baik, 2020). Both hormones can supply sufficient blood to reach the brain, muscles, and lungs to deal with the situation (Privitera et al., 2024). NE plays a role in sensation, cognition, attention, and appetite regulation (Borodovitsyna et al., 2017). In the gastrointestinal tract, the catecholamines increase gastric acid production and disrupt epithelial barrier integrity, which can contribute to mucosal damage and ulcer formation. Activation of the sympathetic nervous system can release NE, resulting in decreased blood flow to the gastric mucosa, thereby impairing its ability to heal and maintain integrity (Sgambato et al., 2016). Additionally, NE can modulate energy intake, thermal homeostasis, and gut motility (Rusch et al., 2023) (Table 1).

**Table 1:** Role of gut microbiota neurotransmitters in prevention stress ulcer.

Category	Item	Mechanism	Mechanism of action	References
Monoamines	Dopamine	Regulates GI motility, blood flow, and mucosal barrier; protects against gastroduodenal ulcers.	Increase in dopamine enhances mucosal protection and reduces ulcer risk.	Liu et al. (2021); Jia et al. (2023); Belujon and Grace (2017)
	Norepinephrine (NE)	Disrupt epithelial barrier integrity; modulates energy intake, thermal homeostasis, and gut motility.	NE increases gastric acid secretion but reduces mucosal blood flow under stress.	Baik (2020); Privitera et al. (2024); Rusch et al. (2023)
	Epinephrine	Ensures energy supply via glycogen and fatty acid mobilization; involved in rapid stress response.	Epinephrine mobilizes energy but may exacerbate mucosal damage under prolonged stress.	Baik (2020); Privitera et al. (2024)
	Serotonin (5-HT)	Regulates sleep, mood, anxiety, and GI motility; increases BBB permeability; stress-induced dysbiosis reduces 5-HT, impairing mucosal integrity.	Decrease in 5-HT reduces mucosal repair and increases ulcer susceptibility.	Strandwitz (2018); Baik (2020); Wan et al. (2020)
Amino acids	Glutamate	Influences learning, memory, and GI motility; stimulates 5-HT secretion; protects mucosa by boosting mucosal defenses.	Glutamate enhances mucosal defenses and reduces acid-induced damage.	Brekke et al. (2016); Bailey and Cryan (2017); Akiba et al. (2009)
	GABA	Regulates heart rate, blood pressure, GI motility, and inflammation; stress-induced dysbiosis reduces GABA, exacerbating inflammation.	Decrease in GABA exacerbates inflammation and mucosal damage.	Woo et al. (2021); Wu and Sun (2015); Szpręgiel et al. (2021)
Glucocorticoids	Cortisol	Long-term stress adaptation; cortisol protects against stress but dysbiosis increases cortisol.	Increased cortisol promotes pathogenic bacterial growth and mucosal damage.	Kageyama et al. (2021); Keskitalo et al. (2021); Périco et al. (2020)
Acetylcholine	Acetylcholine (ACh)	Regulates GI secretion, motility, and enteric neurotransmission; stress-induced dysregulation contributes to ulcers.	Dysregulation of ACh signaling disrupts gut motility and mucosal integrity.	Kageyama et al. (2021); Popovic et al. (2023); Martínez-Augustín et al. (2000)
Neuropeptides	CRH	Slows gastric emptying, stimulates colonic motility, and damages intestinal epithelial barrier.	CRH increases intestinal permeability and mucosal damage.	Kageyama et al. (2021); Rodiño-Janeiro et al. (2015)
	Orexin	Regulates intestinal permeability, and immune cell activation; promotes mucosal regeneration and gastric blood flow for ulcer healing.	Orexin enhances mucosal regeneration and blood flow, promoting ulcer healing.	Couvineau et al. (2021); Grafe and Bhatnagar (2018); Mediavilla (2020)
	Ghrelin	Regulates gastric secretion, and gut motility; aids in ulcer healing through mucosal regeneration and blood flow.	Ghrelin promotes mucosal repair and reduces inflammation.	Akalu et al. (2020); Mediavilla (2020)
	NPY	Released in response to stress; regulates hunger, pain, mood, and memory; has antibacterial and neuroprotective properties.	NPY reduces stress-induced mucosal damage and inflammation.	Zhang et al. (2024); Lach et al. (2018); Henry et al. (2017)
	PYY	Regulates food intake and memory; penetrates BBB to influence brain function.	PYY modulates gut-brain signaling and reduces stress-induced mucosal damage.	Lach et al. (2018); Henry et al. (2017)
	CCK	Regulates pain, cognition, and feeding behavior; accelerates ulcer healing via somatostatin release and hyperemia.	CCK promotes ulcer healing through hyperemia and somatostatin release.	Bauer et al. (2016); West et al. (2003)
	Glucagon-like peptide (GLP)	Inhibits gastric movement and insulin secretion; has anti-inflammatory and anti-apoptotic functions; microbial metabolites increase GLP secretion.	GLP enhances mucosal blood flow and reduces inflammation.	Abdalqadir and Adeli (2022); Diz-Chaves et al. (2020); Zeng et al. (2024)
	VIP	Reduces vascular sensitivity to NE and angiotensin II; protects stomach tissue from lipid peroxidation and ulcers; has anti-inflammatory and antioxidant functions.	VIP reduces oxidative stress and protects mucosal integrity.	Withana and Castorina (2023); Tunçel et al. (1998)

Dopamine functions in motivation, memory, mood, attention, risk assessment, and decision making. After exposure to stress, the dopaminergic reward system must be regulated to monitor and coping mechanism for stressful situations (Belujon and Grace, 2015). Dopamine reduces gut motility, promotes secretion and mucosal blood flow, and protects against gastroduodenal ulcers in the gastrointestinal system (Belujon and Grace, 2017). Stress-induced dysbiosis may decrease dopamine production, thereby affecting GI motility, blood flow, and mucosal barrier function (Baik, 2020) (Table 1).

Most of the 5-HT is formed in the gastrointestinal tract. Gut bacteria stimulate the intestine to secrete 5-HT (Strandwitz, 2018). 5-HT does not cross the BBB, but increases BBB permeability, which indirectly affects brain function. Sleep, anxiety, mood, hunger, sickness, and social and sexual behaviors are all regulated by 5-HT (Baik, 2020). By its anti-immune properties, it stimulates the production of cytokines (Wan et al., 2020). The gastrointestinal tract influences motility and mucus and bicarbonate secretion (Khoder et al., 2016). Under stress, dysbiosis reduces 5-HT levels, impairs mucosal integrity, and enhances vulnerability to inflammation and ulceration (Strandwitz, 2018) (Table 1).

#### Amino-acids

Excitatory glutamate is secreted by brain cells and neurons (Brekke et al., 2016). Acute stress stimulates glutamate secretion by activating glucocorticoid receptors (Pal, 2021). Amino acids functions are regulation of learning, memory, appetite (Bailey and Cryan, 2017), gastrointestinal motility, endocrine function, mucus, and bicarbonate secretion. Furthermore, glutamate stimulates 5-HT secretion by enteroendocrine cells (San Gabriel and Uneyama, 2013). L-glutamate preperfusion stopped acid-induced cellular damage, indicating that L-glutamate shields the mucosa by boosting mucosal defenses (Akiba et al., 2009).

In this context, “metabolic activity of NMDA” refers to the expression levels, membrane localization, and functional responsiveness of NMDA receptors, which are influenced by the gut microbiota. Dysbiosis—particularly antibiotic-induced—has been shown to reduce NMDA receptor expression and alter receptor trafficking, thereby impairing glutamatergic signaling relevant to gut-brain axis function (Bailey and Cryan, 2017).

Parabacteroides, Eubacterium, and Bifidobacterium produce GABA (Woo et al., 2021). In addition to controlling heart rate and blood pressure, GABA is essential for several gastrointestinal tract processes, including inflammation, motility, gastric emptying (Wu and Sun, 2015), immunological responses, anxiety, depressive symptoms, and pain perception (Chen et al., 2021). Stress-induced dysbiosis may reduce GABA levels, impair the mucosal barrier, and exacerbate inflammation in the GI tract (Szpręgiel et al., 2021). Reduced GABA signaling under stress can lead to heightened anxiety and stress responses, which may exacerbate gastric mucosal damage and increase ulcer risk (Strandwitz, 2018) (Table 1).

#### Glucocorticoids

This is a type of long-term stress adaptation (Kageyama et al., 2021). Normal cortisol secretion protects the body from stress primarily by regulating glucose metabolism and enhancing the vascular response to catecholamines. While it has some influence on fluid balance, the regulation of salt and water homeostasis is primarily mediated by aldosterone through activation of mineralocorticoid receptors (Keskitalo et al., 2021). When dysbiosis causes cortisol levels to increase, mast cells may get activated. Heparin, histamine, proinflammatory cytokines, proteases, and tryptase can all be released by these granules. Histamine is essential in the pathogenesis of stress-related diseases and activation of the H2 receptor in parietal cells (Kageyama et al., 2021). Cortisol also alters the gut environment, promoting the growth of pathogenic bacteria over beneficial bacteria (Mediavilla, 2020) (Table 1).

#### Acetylcholine (ACh)

ACh helps in both immediate physiological reactions to stress and memories of stressful situations that may have an impact on long-term behavioral patterns (Mineur and Picciotto, 2021). It also regulates gastrointestinal tract secretion, motility, enteric neurotransmission, stress coding, memory, and cognition (Withana and Castorina, 2023). Stress can lead to dysregulation of ACh signaling, resulting in gut motility issues and contributing to the development of ulcers (Kageyama et al., 2021).

#### Neuropeptides

Enteroendocrine cells in the gut produce a variety of neuropeptides that play essential roles in maintaining gastrointestinal and neuroimmune homeostasis. These neuropeptides, including Neuropeptide Y (NPY), Peptide YY (PYY), cholecystokinin (CCK), substance P, glucagon-like peptides (GLP-1 and GLP-2), and vasoactive intestinal peptide (VIP), act locally within the enteric nervous system or systemically via the bloodstream (Cani and Knauf, 2016). Their activity is significantly influenced by gut microbial metabolites, particularly during stress (Iftikhar et al., 2020).

Under moderate stress, CRH is used to adjust humoral and behavioral reactions and memory (Kageyama et al., 2021). Severe stress causes hyperexcitability and seizures (Leistner and Menke, 2020). In the gastrointestinal tract, colon and ileum cells are the primary sources of CRH secretion (Liu et al., 2016). According to Rodiño-Janeiro et al. (2015), CRH slows gastric emptying, stimulates colonic motility, and damages the intestinal epithelial barrier. These effects are not reliant on stressful environments (Yang et al., 2016).

Excitatory neuropeptides known as orexins or hypocretins are produced from the prepro-orexin precursor and are found in cells located in the lateral and posterior hypothalamus regions. In both humans and animals, orexin has also been found in the enteroendocrine gut cells, enteric nervous system, and neurons and mucosa of every area of the gut (Couvineau et al., 2021). The stomach produces and releases the hunger hormone ghrelin, with minor amounts originating from the brain, pancreas, and small intestine. Orexin can pass through the blood–brain barrier (BBB) (Akalu et al., 2020).

Orexin and ghrelin have been shown to regulate stress responses. Acute behavioral and neuroendocrine responses to stress are enhanced by orexin. Orexin controls intestinal permeability, inhibits immune cell activation, and protects against central and systemic inflammation (Grafe and Bhatnagar, 2018). Ghrelin plays a role in reward processes, mood, memory, learning, and stress responses. It plays a role in stimulating gastric and pancreatic secretion and gut motility (Akalu et al., 2020).

Together with nitric oxide and the vasoactive neuropeptide calcitonin gene-related peptide, ghrelin and orexin aid in the healing of chronic stomach ulcers. Orexin functions through the vagal pathway to provide protection. Additionally, orexin is crucial for healing chronic gastric ulcers because it promotes mucosal regeneration and gastric blood flow at the ulcer margin (Mediavilla, 2020).

The control of hunger, pain, emotion, mood, cognition, stress, intake, and energy balance is all affected by NPY (Zhang et al., 2024). NPY is also produced in the gastrointestinal tract (Lach et al., 2018). PYY is mostly secreted by colon and ileum cells. Pancreatic polypeptides are produced by the vagus nerve and released in response to food. Using transmembrane diffusion, PYY and pancreatic polypeptide may both penetrate the BBB and attach to cognate receptors in the postrema region (Henry et al., 2017). YPY is formed in the brain, from the medulla to the cortex. Its receptors (Y1, Y2, Y3, Y4, and Y5) are expressed in brain neurons, gut primary afferents, immunological cells, sympathetic neurons, and the hypothalamus (Lach et al., 2018). The NPY family has antibacterial, neuroprotective, neurogenic, and neuroinflammatory properties and controls blood pressure, food intake, and memory (Henry et al., 2017) (Table 1).

Cholecystokinin (CCK) is a peptide hormone released by I cells in the duodenum and jejunum in response to fatty acids and amino acids (Bauer et al., 2016). It regulates gallbladder contraction, pancreatic enzyme secretion, gastric emptying, and also affects pain perception, mood, anxiety, and satiety (Wang et al., 2020). CCK contributes to mucosal defense by stimulating somatostatin release, enhancing blood flow (hyperemia), and promoting sensory nerve activation at ulcer sites. Gut microbiota composition has been shown to influence CCK levels, suggesting microbial modulation of host satiety and healing responses (West et al., 2003) (Table 1).

Glucagon-like peptide 1 (GLP-1) and GLP-2 are secreted by intestinal L cells and are heavily regulated by microbial metabolites such as SCFAs (Abdalqadir and Adeli, 2022). GLP-1 enhances glucose-stimulated insulin secretion, inhibits gastric motility, and suppresses appetite, while also exhibiting anti-inflammatory and anti-apoptotic properties (Diz-Chaves et al., 2020). GLP-2 promotes intestinal epithelial proliferation, enhances mucosal blood flow (Stenman et al., 2015) and supports barrier integrity through modulation of the linoleic acid metabolic pathway (Zeng et al., 2024). Together, these peptides form a protective axis against stress-induced gastrointestinal damage (Zhang et al., 2022) (Table 1).

Substance P is an excitatory neuropeptide that mediates neurogenic inflammation, pain, and immune cell activation (Cani and Knauf, 2016). It is elevated in the gut during psychological or physical stress and is associated with increased intestinal permeability, mast cell degranulation, and mucosal inflammation (Iftikhar et al., 2020). Overexpression of substance P can exacerbate stress-related mucosal injury by promoting proinflammatory cytokine release and epithelial barrier breakdown.

Vasoactive intestinal peptide (VIP) is a neuropeptide found in enteric neurons and gut mucosa. It is a potent vasodilator and immunomodulator, involved in regulating intestinal motility, smooth muscle relaxation, secretion of digestive fluids, and anti-inflammatory responses (Withana and Castorina, 2023). VIP is known to protect the gastric mucosa from oxidative damage and inhibit mast cell degranulation, thus reducing stress-related ulceration. Its levels are influenced by gut microbiota and its actions are modulated through interactions with glial and immune cells in the enteric nervous system (Tunçel et al., 1998) (Table 1).

### Potential therapeutic strategies for stress ulcer targeting the microbiota-brain-gut axis

#### Dietary modification

In critically ill patients, stress ulcers are common due to prolonged mechanical ventilation, hemodynamic instability, and the use of medications that affect gastric mucosal integrity. Dietary strategies should focus on minimizing acid hypersecretion, supporting mucosal protection, and reducing systemic inflammation (Sheneni et al., 2023).

The Mediterranean diet, known for its antioxidant, anti-inflammatory, and neuroprotective properties, may benefit critically ill patients by enhancing gut microbial diversity and short-chain fatty acid (SCFA) production. However, modifications are needed to accommodate enteral feeding and patient-specific nutritional needs (Sofi et al., 2013). High-fiber diets, including fruits (apple, banana, mango, melon, and papaya), vegetables (spinach, carrot, bean, beet, kale, and leek) (Almahal et al., 2025), cereals (brown rice, bulgur, millet, oatmeal), and legumes (bean soup, lentils, chickpeas, and soybean), promote mucin formation and gut barrier integrity. Fiber and essential fatty acids help mitigate stress-induced peptic ulcers and support immune function (Kulshreshtha et al., 2017).

For critically ill patients, diets combining elements of the Mediterranean and Dietary Approaches to Stop Hypertension (DASH) diets, rich in vegetables, whole grains, fruits, low-fat dairy, and lean protein, may offer additional protective effects against ulcer formation and improve clinical outcomes (Prasad, 2009). Micronutrients such as zinc, selenium, vitamin C, *β*-carotene, and vitamin E play critical roles in wound healing and oxidative stress reduction. Zinc promotes mucosal repair and immune function (Prasad, 2009), selenium enhances ulcer healing and infection control, and β-carotene and vitamin C contribute to gastric mucosal protection (Kulshreshtha et al., 2017). Vitamin E aids in ulcer treatment and enhances mucosal recovery (Yousaf et al., 2014).

Intermittent fasting may have potential benefits in critically ill patients by modulating gut microbiota, reducing systemic inflammation, and decreasing gastric acid secretion. However, its application requires careful consideration in ICU settings (Paoli et al., 2019).

#### Prebiotics

Prebiotics support gut health by promoting the growth of beneficial bacteria, which is particularly crucial for critically ill patients at high risk of gut dysbiosis (Al-garni et al., 2021).

Yeast beta-glucan enhances SCFA production, restores gut microbiotabalance, and reduces systemic and neuroinflammation. It also promotes epithelial hyperplasia, ulcer healing, fibroblast proliferation, and angiogenesis (Medeiros et al., 2012).

Mannan oligosaccharides stimulate SCFA synthesis while reducing oxidative stress and pro-inflammatory cytokines (TNF-α, IL-1 and IL-6), thereby protecting the gastric mucosa in critically ill patients (Ashaolu, 2020).

Lactulose plays a crucial role in modulating gut microbiota composition, reducing inflammation, and improving insulin sensitivity. Additionally, it inhibits inflammatory carcinogenesis and restores intestinal barrier integrity (Hiraishi et al., 2022).

Ferulic acid exerts potent anti-inflammatory and antioxidant effects, supporting nerve growth factor production and reinforcing gastric mucosal integrity. By blocking neutrophil infiltration and lipid peroxidation, lactulose has gastroprotective properties that are particularly relevant in ICU patients with stress ulcers (Ermis et al., 2023). Despite these benefits, further research is necessary to standardize prebiotic use in clinical practice, considering individual factors such as diet, age, and comorbidities (Barbosa and Vieira-Coelho, 2020) (Table 2).

**Table 2:** Possible treatment approaches for stress ulcers that focus on the gut-brain-microbiota axis.

Category	Item	Mechanism	Notes	References
Prebiotics	Yeast beta-glucan	Increases SCFA production; balances anti- and pro-inflammatory bacteria; reduces neuroinflammation.	Promotes ulcer healing, fibroblast proliferation, and angiogenesis.	Medeiros et al. (2012)
	Mannan oligosaccharides	Enhances SCFA synthesis; reduces oxidative stress and pro-inflammatory cytokines (TNF-α, IL-6, IL-1).	Reduces gastric injury and inflammation.	Ashaolu (2020)
	Lactulose	Reduces neuroinflammation; promotes insulin sensitivity; restores gut microbiota composition.	Inhibits inflammatory carcinogenesis and reduces inflammation.	Hiraishi et al. (2022)
	Ferulic acid	Anti-inflammatory and antioxidant effects; increases nerve growth factor and brain-derived neurotrophic factor; blocks NF-κB, reducing tissue damage.	Protects gastric mucosa and maintains structural integrity.	Ermis et al. (2023)
Probiotics	*Lactobacillus* & *Bifidobacterium*	Modulates immune response; reduces inflammation and reactive oxygen species; enhances antioxidant enzymes and inhibits apoptosis.	Stabilizes mast cells and protects gastric mucosa from stress-induced damage.	Paoli et al. (2019); Al-garni et al. (2021)
Fecal microbiota transplantation (FMT)	FMT	Restores gut microbial diversity and SCFA production; repairs gut barrier and restores secondary bile acid metabolism.	Administered via colonoscopy, enema, or capsule; safety concerns require further research.	Allegretti et al. (2019); Mullish et al. (2018); Khoruts and Sadowsky (2016)

#### Probiotics

Probiotics modulate the gut microbiota toward a favorable balance, making them an essential adjunct therapy for stress ulcers in critically ill patients (Al-garni et al., 2021). *Lactobacillus and Bifidobacterium,* the most widely studied probiotics, regulate the host immune response, reduce inflammation, prevent pathogen overgrowth, and enhance antioxidant enzyme activity (Paoli et al., 2019; Al-garni et al., 2021).

In critically ill patients, probiotics help restore mucosal integrity by inhibiting apoptosis, stabilizing mast cells, and preventing excessive activation of the hypothalamic–pituitary–adrenal (HPA) axis, which contributes to gastric mucosal protection (Mal et al., 2024). Combining probiotics with prebiotics enhances their therapeutic potential, promoting epithelial cell proliferation, particularly at ulcerated margins (You et al., 2022).

Probiotic and prebiotic therapies have shown efficacy in reducing oxidative stress, pro-inflammatory cytokines, and gastric mucosal injury. Studies suggest that probiotics may be the most effective therapeutic group for stress ulcer prevention in ICU patients (Al-garni et al., 2021). However, the harsh physiological conditions in critically ill patients, including acidic gastric pH, mechanical stress, and digestive enzymes, may limit probiotic colonization in the gut. Further research is needed to optimize probiotic strains, dosages, and delivery methods tailored to ICU settings (Allegretti et al., 2019) (Table 2).

#### Fecal microbiota transplantation (FMT)

FMT is an emerging therapy for restoring gut microbiota diversity and function in critically ill patients with severe dysbiosis (Allegretti et al., 2019). It involves transplanting prescreened donor feces via colonoscopy, enema, or capsule to enhance SCFA production and reestablish gut microbial homeostasis (Mullish et al., 2018).

In critically ill patients, FMT may support gut barrier repair, regulate mucosal immune responses, and restore secondary bile acid metabolism, which plays a crucial role in gastrointestinal health (Khoruts and Sadowsky, 2016). However, concerns regarding safety, infection risk, and treatment standardization require further investigation before widespread clinical implementation (Feng et al., 2023) (Table 2).

## Conclusion and future perspectives

Stress ulcers develop through a complex interplay of physiological stress, neurohormonal activation, and weakened mucosal defense. This review has emphasized the central role of the gut microbiota-brain-gut axis in modulating these processes. Under stress, colonic dysbiosis—characterized by a loss of microbial diversity and altered metabolite production—triggers systemic immune activation and disrupts neuroenteric signaling. These changes reduce the availability of protective microbial metabolites like short-chain fatty acids (SCFAs) and tryptophan derivatives, impair vagal tone, and increase circulating proinflammatory cytokines. Collectively, these responses affect upper gastrointestinal physiology by promoting gastric acid hypersecretion, reducing mucosal blood flow, and compromising mucus and bicarbonate secretion—conditions conducive to stress ulcer formation.

Although the stomach and duodenum contain few resident microbes, they remain highly responsive to downstream effects of colonic dysbiosis via the microbiota–immune–neuroendocrine axis. Correcting this dysbiosis through dietary interventions, prebiotics, probiotics, or fecal microbiota transplantation helps restore microbial balance, reduce inflammation, and reestablish mucosal integrity. These therapies indirectly enhance gastric protection by stabilizing systemic stress responses and reinforcing upper GI defense mechanisms. Future research should focus on identifying specific microbial signatures and molecular mediators that can be therapeutically targeted to prevent or mitigate stress-induced gastroduodenal injury.
